# Supplementary material for: Systematic generation of biophysically detailed models for diverse cortical neuron types
Source: Nat Commun. 2018 Feb 19;9:710. doi: 10.1038/s41467-017-02718-3 (PMC5818534; doi:10.1038/s41467-017-02718-3)
Supplement: Supplementary file 1 — Supplementary Information [file 41467_2017_2718_MOESM1_ESM.docx]

Supplementary information for “Systematic generation of biophysically-detailed models for diverse cortical neuron types”

Nathan W. Gouwens, Jim Berg, David Feng, Staci A. Sorensen, Christof Koch, Anton Arkhipov

# SUPPLEMENTARY METHODS

### Mechanism definitions

Most active conductances were defined using the Hodgkin-Huxley formalism. When equations for rate constants (e.g., $\alpha_{n}$, $\beta_{n}$) are given, the steady state values were calculated as $n_{\infty}=\alpha_{n}/(\alpha_{n}+\beta_{n})$ and the time constant values were calculated as $\tau_{n}=1/(\alpha_{n}+\beta_{n})$, unless otherwise indicated. Rate constants were scaled from the temperatures of the original study to match our experimental temperature of 34 °C with a $Q_{10}$ of 2.3. Rate constants are in units of ms^-1^, time constants are in units of ms, and voltages are in units of mV.

**High-voltage-activated calcium current (Ca_HVA)**^1^: No temperature scaling was applied.

$$g=\overline{g}m^{2}h (1)$$

$$\alpha_{m}=0.055\frac{-27-V}{\exp\left( \frac{-27-V}{3.8} \right)-1} (2)$$

$$\beta_{m}=0.94\frac{-75-V}{\exp\left( \frac{-75-V}{17} \right)-1} (3)$$

$$\alpha_{h}=4.57\times{10}^{-4}\exp\left( \frac{-13-V}{50} \right) (4)$$

$$\beta_{h}=\frac{6.5\times{10}^{-3}}{\exp\left( \frac{-15-V}{28} \right)+1} (5)$$

**Low-voltage-activated calcium current (Ca_LVA)**^2,3^: Time constants scaled from 21 °C. Additional +10 mV junction potential correction applied.

$$g=\overline{g}m^{2}h (6)$$

$$m_{\infty}=\frac{1}{1+\exp\left( \frac{V+30}{-6} \right)} (7)$$

$$\tau_{m}=5+\frac{20}{1+\exp\left( \frac{V+25}{5} \right)} (8)$$

$$h_{\infty}=\frac{1}{1+\exp\left( \frac{V+80}{6.4} \right)} (9)$$

$$\tau_{h}=20+\frac{50}{1+\exp\left( \frac{V+40}{7} \right)} (10)$$

**Intracellular Ca^2+^ dynamics**^4^: Changes in the internal calcium concentration due to transmembrane calcium currents ($I_{\mathrm{Ca}}$) were modeled in a 100 nm submembrane shell with a buffer and extrusion pump. The dynamics followed the equation:

$$\frac{d[\mathrm{Ca}^{2+}]_{i}}{dt}=\frac{I_{\mathrm{Ca}}\gamma}{2Fd}-\frac{[\mathrm{Ca}^{2+}]_{i}-[\mathrm{Ca}^{2+}]_{i,min}}{\tau_{\mathrm{decay}}} (11)$$

where $d$ is the depth of the shell, $\gamma$ is the fraction of free calcium, $\tau_{\mathrm{decay}}$ is the time constant of removal, $F$ is Faraday’s constant, and $[\mathrm{Ca}^{2+}]_{i,min}$ = 0.0001 mM.

**Hyperpolarization-activated cation current (Ih)**^5^: No temperature scaling was applied. Reversal potential was $-45$ mV.

$$g=\overline{g}m (12)$$

$$\alpha_{m}=0.00643\frac{V+154.9}{\exp\left( \frac{V+154.9}{11.9} \right)-1} (13)$$

$$\beta_{m}=0.193\exp(V/33.1) (14)$$

**M current (Im) - set A**^6^: Time constants scaled from 21 °C.

$$g=\overline{g}m (15)$$

$$\alpha_{m}=0.0033\exp(0.1(V+35)) (16)$$

$$\beta_{m}=0.0033\exp(-0.1(V+35)) (17)$$

**M current (Im_v2) - set B**^7^: Time constants scaled from 30 °C.

$$g=\overline{g}m (18)$$

$$\alpha_{m}=0.007\exp(2.4(V+48)/26.12) (18)$$

$$\beta_{m}=0.007\exp(-3.6(V+48)/26.12) (19)$$

$$\tau_{m}=15+\frac{1}{\alpha_{m}+\beta_{m}} (20)$$

**Slow, persistent K current (Kp) - set A**^8^: Time constants scaled from 21 °C.

$$g=\overline{g}m^{2}h (21)$$

$$m_{\infty}=\frac{1}{1+\exp\left( \frac{-(V+14.3)}{14.6} \right)} (22)$$

$$\tau_{m}= \left\{ \begin{aligned} 1.25+175.03\exp\left( 0.026 V \right) \text{if }V< -50 \\ 1.25+13\exp\left( -0.026 V \right) \text{if }V\geq-50 \end{aligned} \right. (23)$$

$$h_{\infty}=\frac{1}{1+\exp\left( \frac{V+54}{11} \right)} (24)$$

$$\tau_{h}=360+(1010+24(V+55))\exp\left( -\left( \frac{V+75}{48} \right)^{2} \right) (25)$$

**Kv1-like current (Kd) - set B**^9^: Time constants scaled from 23 °C.

$$g=\overline{g}mh (26)$$

$$m_{\infty}=1-\frac{1}{1+\exp\left( \frac{v+43}{8} \right)} (27)$$

$$\tau_{m}=1 (28)$$

$$h_{\infty}=\frac{1}{1+\exp\left( \frac{V+67}{7.3} \right)} (29)$$

$$\tau_{h}=1500 (30)$$

**Kv2-like current (Kv2like) - set B**^10,11^: Time constants scaled from 21 °C.

$$g=\overline{g}m^{2}\frac{h_{1}+h_{2}}{2} (31)$$

$$\alpha_{m}=0.048\frac{-(V-43}{\exp\left( \frac{-(V-43)}{11} \right)-1} (31)$$

$$\beta_{m}=0.008\exp\left( \frac{-(V+1.27)}{120} \right) (32)$$

$$h_{1,\infty}=h_{2,\infty}=\frac{1}{1+\exp\left( \frac{V+58}{11} \right)} (33)$$

$$\tau_{h_{1}}=360+(1010+24(V+55))\exp\left( -\left( \frac{V+75}{48} \right)^{2} \right) (33)$$

$$\tau_{h_{2}}=2350+1380\exp(-0.011V)-210\exp(-0.03V) (34)$$

**Kv3-like current (Kv3_1)**^12^: No temperature scaling was applied.

$$g=\overline{g}m (35)$$

$$m_{\infty}=\frac{1}{1+\exp\left( -\frac{V-18.7}{9.7} \right)} (36)$$

$$\tau_{m}=\frac{0.01}{1+\exp\left( -\frac{V+46.56}{44.14} \right)} (37)$$

**Fast, transient K current (Kt)**^8^: Time constants scaled from 21 °C.

$$g=\overline{g}m^{4}h (38)$$

$$m_{\infty}=\frac{1}{1+\exp\left( \frac{-(V+47)}{29} \right)} (38)$$

$$\tau_{m}=0.34+0.92\exp\left( -\left( \frac{V+71}{59} \right)^{2} \right) (39)$$

$$h_{\infty}=\frac{1}{1+\exp\left( \frac{V+66}{10} \right)} (40)$$

$$\tau_{h}=8+49\exp\left( -\left( \frac{V+73}{23} \right)^{2} \right) (41)$$

**Small conductance Ca-activated K current**^13^: No temperature scaling was applied.

$$g=\overline{g}z (42)$$

$$z_{\infty}=\frac{1}{1+(0.00043/[\mathrm{Ca}^{2+}]_{i})^{4.8}} (43)$$

$$\tau_{z}=1 (44)$$

**Persistent Na current (NaP) - set A**^14^: Time constants scaled from 21 °C.

$$g=\overline{g}m_{\infty}h (45)$$

$$m_{\infty}=\frac{1}{1+\exp\left( -\frac{V+52.6}{4.6} \right)} (46)$$

$$h_{\infty}=\frac{1}{1+\exp\left( \frac{V+48.8}{10} \right)} (47)$$

$$\alpha_{h}=2.88\times{10}^{-6}\frac{V+17}{\exp\left( \frac{V+17}{4.63} \right)-1} (48)$$

$$\beta_{h}=6.94\times{10}^{-6}\frac{-(V+64.4)}{\exp\left( \frac{-(V+64.4)}{2.63} \right)-1} (49)$$

**Transient Na current (NaT) - set A**^15^: Time constants scaled from 23 °C.

$$g=\overline{g}m^{3}h (50)$$

$$\alpha_{m}=0.182\frac{-(V+40)}{\exp\left( \frac{-(V+40)}{6} \right)-1} (51)$$

$$\beta_{m}=0.124\frac{V+40}{\exp\left( \frac{V+40}{6} \right)-1} (52)$$

$$\alpha_{h}=0.015\frac{-(V+66)}{\exp\left( \frac{-(V+66)}{6} \right)-1} (53)$$

$$\beta_{h}=0.015\frac{V+66}{\exp\left( \frac{V+66}{6} \right)-1} (54)$$

**Voltage-dependent Na current (NaV) - set B**^16^: Time constants scaled from 37 °C.

$$g=\overline{g}O (55)$$

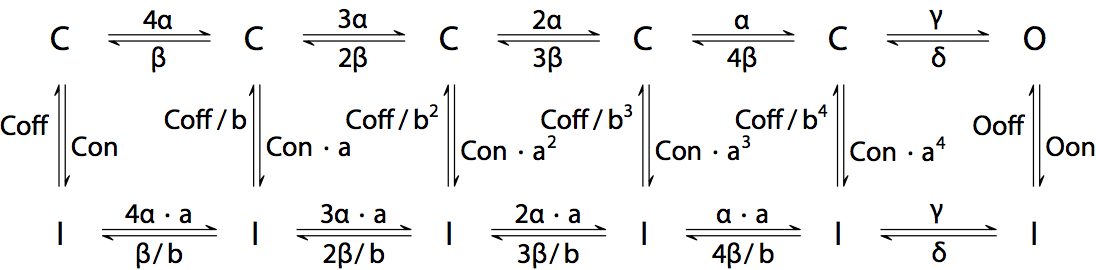
(56)

where Con = 0.01 ms^-1^, Coff = 40 ms^-1^, Oon = 8 ms^-1^, Ooff = 0.05 ms^-1^, $\alpha=400\exp(V/24)$ ms^-1^, $\beta=12\exp(-V/24)$ ms^-1^, $\gamma$ = 250 ms^-1^, $\delta$ = 60 ms^-1^, a = 2.51, and b = 5.32.

### Passive parameters estimates with morphological corrections

The effects of altering the reconstructed cell morphologies on the passive parameter estimates from direct fitting (see Methods) were investigated. Only aspiny cells (n = 66) were analyzed so that an additional scaling factor to account for the presence of dendritic spines, which were not explicitly represented in the morphologies, would not be necessary.

Shrinkage of the tissue in the z-dimension (i.e., orthogonal to the cutting plane of the slice) was corrected for by expanding the lengths of the processes in that direction by two- or three-fold, then re-fitting the adjusted morphology (Supplementary Fig. 1b). The diameters were not changed since diameter estimates were based only on the higher-resolution x- and y-dimensions (0.114 µm per pixel; z-dimension 0.28 µm per pixel) of the images. The tissue was estimated to have shrunk to ~30% of its original value by comparing the distance from the soma to the surface of the slice during the recording and after processing in a subset of cells, indicating that the 3x scaling in the z-dimension would be expected to restore the morphology to near its original extent during the recording. The 3x z-scale correction shifted the median *C*_m_ estimate from 2.8 µF cm^-2^ to 1.9 µF cm^-2^ (Fig. 1b).

A subset of the aspiny cells (n = 22) had axonal reconstructions available (Supplementary Fig. 1a). We performed additional passive fits on the morphologies that included the axons (Supplementary Fig. 1c). Doing so shifted the median *C*_m_ estimate of this subset from 2.4 µF cm^-2^ to 1.1 µF cm^-2^, which is near the typically reported value of 1 µF cm^-2^. Including the axon and also applying a 3x z-scale correction shifted the median further downward to 0.8 µF cm^-2^.

### Comparison to models with simplified morphologies

Since the models described in the main text had a soma with active conductances connected to a passive dendritic tree, we tested whether a model without a detailed dendritic morphology could reproduce the firing patterns of the original cells as well, and how much that simplification could speed up the simulation times of the models. We constructed a simplified ball-and-stick morphology for a subset of the cells (n = 26), optimized them in the same way as the models with detailed morphologies, and compared their resulting training errors and simulation times with the models from the main set fit on the same cells.

We fit the responses of the cells to hyperpolarizing one-second-long current steps with a double exponential function to estimate the time constants and coefficients of the charging curve, as well as the input resistance of the cell. These values were used to estimate the dendritic to somatic conductance ratio ($\rho$) and the length (*L*) of the dendritic “stick” in units of length constant^17^, and the dimensions of the ball-and-stick morphology was determined from these parameters and the assumption that the specific membrane capacitance was 1 µF cm^-2^.

Models were fit by placing the same active conductances at the soma (“ball”) of the simplified morphology. The dendritic “stick” remained passive, but the value of the leak conductance was varied during optimization. The optimization procedure was identical to that used in the main text. The best-fit model parameters were identified at the end of optimization and used for comparison with the detailed-morphology models.

Models fit using the ball-and-stick representation typically had higher training errors than the models fit with the detailed morphologies (Fig. 2a) with a median difference in training error of +106%. While some ball-and-stick models fit the original cells as well as the models with detailed morphologies, in general replacing the detailed models with the ball-and-stick versions would lead to a loss of accuracy. Still, the simplified models did, as expected, reduce the simulation times of the models (Fig. 2b) with a median reduction of running time of -53%. In addition, since the optimization procedure used here was developed for the detailed morphology models, it is possible that adjusting the paramaters of the optimization could lead to improvements in the accuracy of the ball-and-stick versions. If faster execution times of biophysical simulations is a goal, this could be a productive avenue for future investigation.

### Comparison of experimental and model features across classes

We examined how electrophysiological features varied across different classes used for prediction analyses (see Fig. 7) and whether model features exhibited similar variation across those classes. In general, features varied with a consistent pattern across transgenic line-based classes (Supplementary Fig. 3), whether they were measured from the original cells or from the models based on those cells. Distributions of features by class were similar between experiments and models, which was not unexpected since we observed relatively low feature errors in our optimized models (see Fig. 5). We found similar results when looking across classes defined by unsupervised clustering (Supplementary Fig. 4).

While we observed similar accuracy scores when attempting to predict these classes through supervised classification with cross-validation using either experimental features or model features (see Fig. 7), it is possible that the classifier trained on model features could be using differences in the model feature space that are not observed in the experimental feature space. For example, if models in the *Pvalb* class erroneously had much wider spikes than other cells (as opposed to actual *Pvalb* cells, which have quite narrow spikes), a classifier would still reliably distinguish *Pvalb* cells based on model features, despite our model set failing to preserve the class differences observed in the original experiments.

Therefore, we investigated whether a classifiers trained to distinguish classes using the experimental features could still perform well when tested using the model features (Supplementary Fig. 5). We found that the classifiers did accurately predict transgenic line-based classes and unsupervised clustering-based classes (76% and 74% testing accuracies, respectively; training accuracies were 90% and 92%). These results indicate that differences in features used by the classifiers remain similar in our model set. We note that the accuracy scores here are not directly comparable to those presented in Fig. 7, which used cross-validation to calculate the testing accuracies of those classifiers; cross-validation was not necessary in this case as we trained and tested on different feature sets.


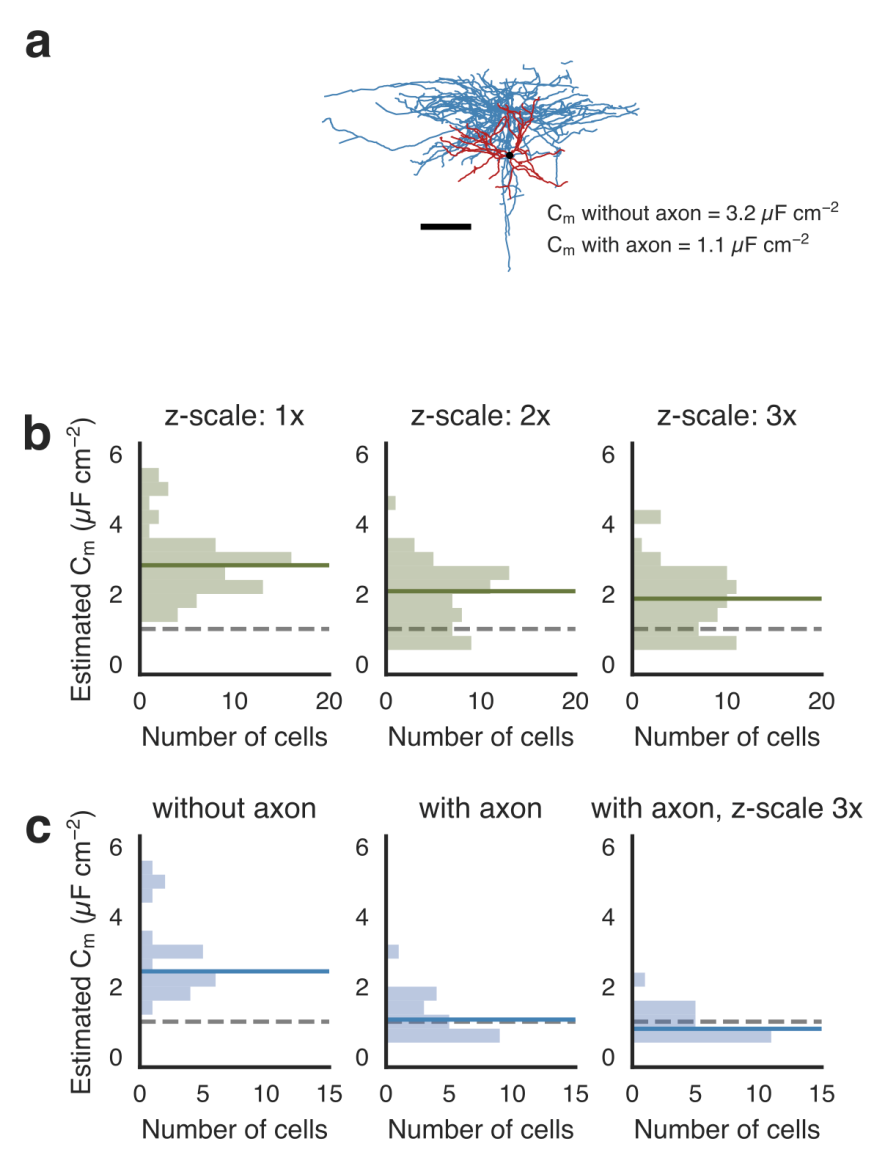


**Supplementary Figure 1:** Assessing effects on C_m_ estimates in aspiny neurons. (**a**) Morphology of an aspiny neuron with its dendrites (blue) and axon (red) reconstructed. The estimated C_m_ value from fitting subthreshold responses with a passive model shifted from 3.2 to 1.1 µF cm^-2^ when the axon was included. Scale bar, 100 µm. (**b**) Effect of compensating for z-shrinkage on estimates of C_m_. The estimates across cells were shifted to lower values when the morphology was expanded (by 2x, middle, and 3x, right) to compensate for z-shrinkage during tissue processing. (**c**) For the subset of cells that had an axonal reconstruction available (left), including the axon during the passive fit shifted the C_m_ estimates to near 1 µF cm^-2^ (middle). Compensating for z-shrinkage reduced the estimates slightly further (right). In both **b** and **c**, median values are indicated by solid colored lines, and the dashed gray line indicates C_m_ = 1 µF cm^-2^.


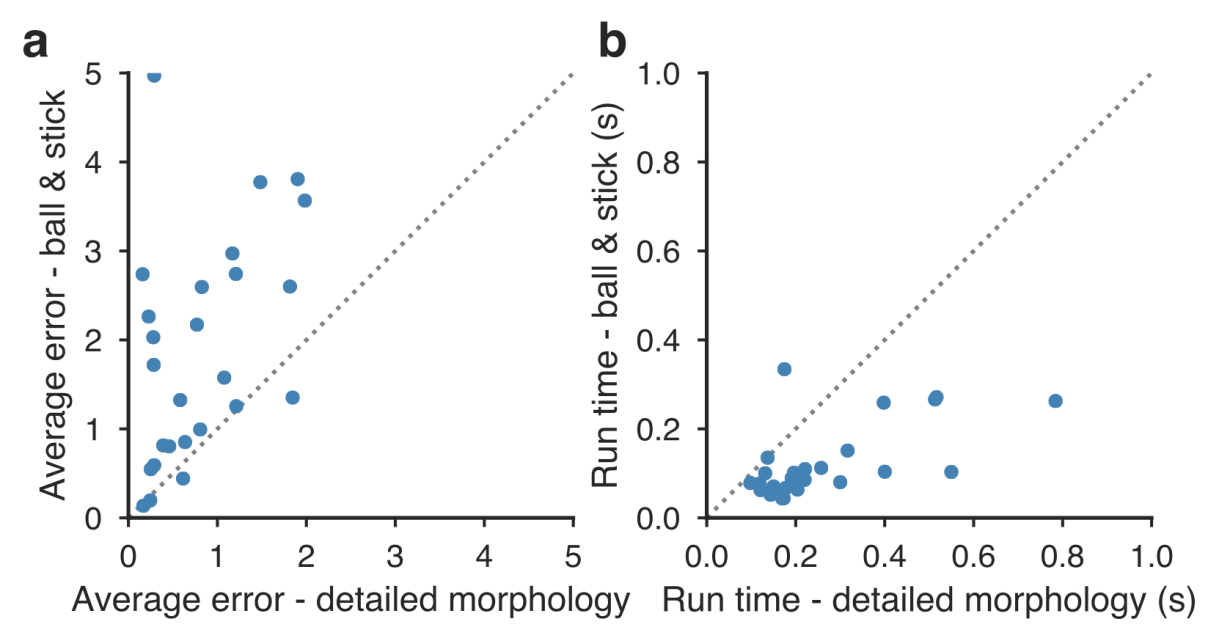


**Supplementary Figure 2:** Comparing models with detailed morphologies to simplified ball-and-stick fits. (**a**) Comparison of average feature errors at the end of optimization for cells fit with both the detailed morphology and a simplified ball-and-stick morphology. (**b**) Comparison of simulation times, using the variable time step method of NEURON, between detailed-morphology models and ball-and-stick models. The protocol simulated was one second with no current injection, followed by a one-second step current injection (set to the amplitude at which the models were each trained), followed by another second with no current injection.


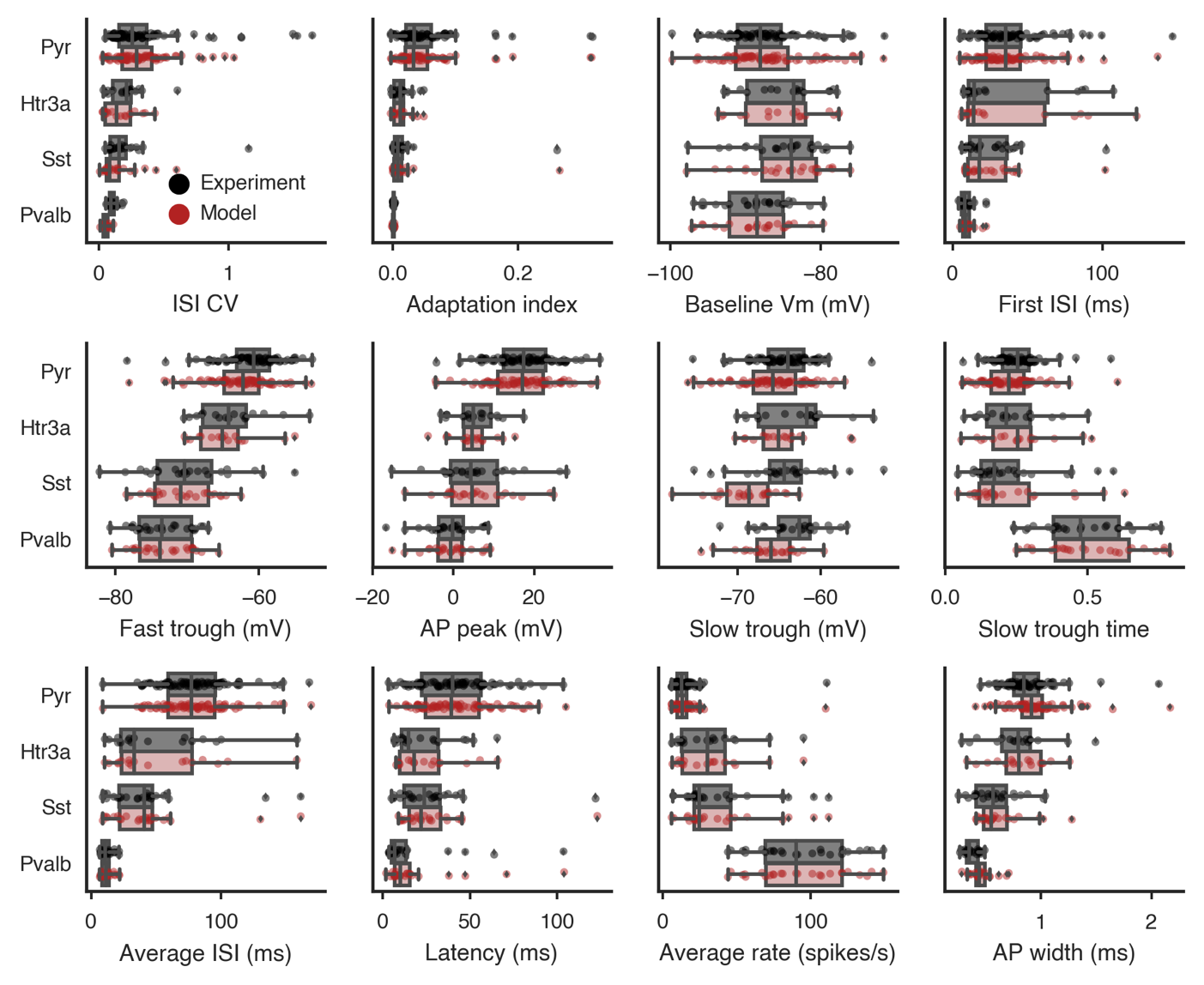


**Supplementary Figure 3:** Comparison of experimental and model features by transgenic group. Boxplots of experimental features (*black*) and model features (*red*) by major transgenically-labeled classes (n=146 cells). Individual data points are also plotted (*circles*). Note that all excitatory-dominant transgenic lines were combined into the “Pyr” category.


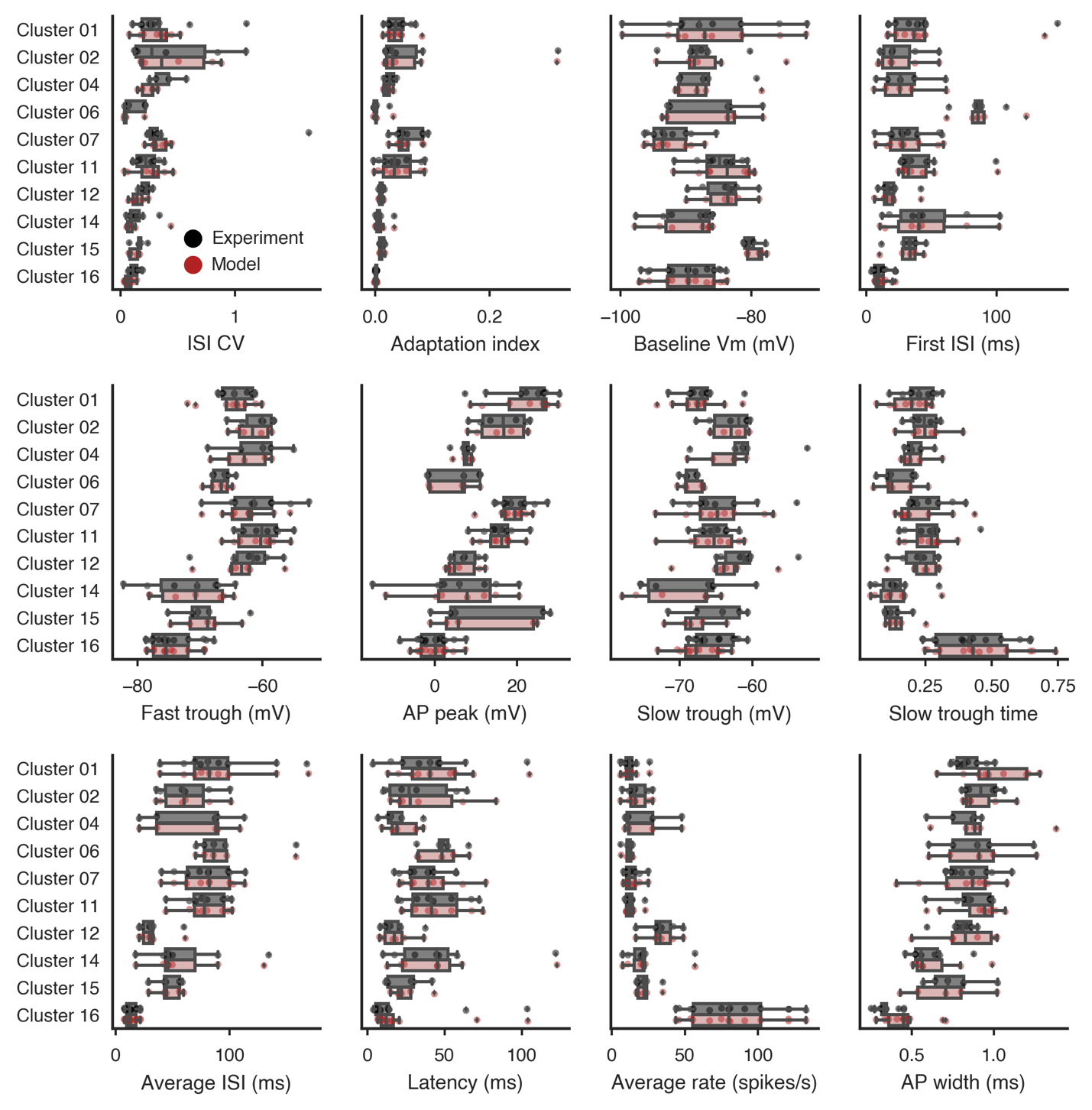


**Supplementary Figure 4:** Comparison of experimental and model features by cluster. Boxplots of experimental features (*black*) and model features (*red*) by class based on unsupervised clustering analysis from Ref. 18. Individual data points are also plotted (*circles*). Only clusters with at least five cells and models were included in the analysis (n=89 cells from 10 out of 16 clusters identified in Ref. 18).


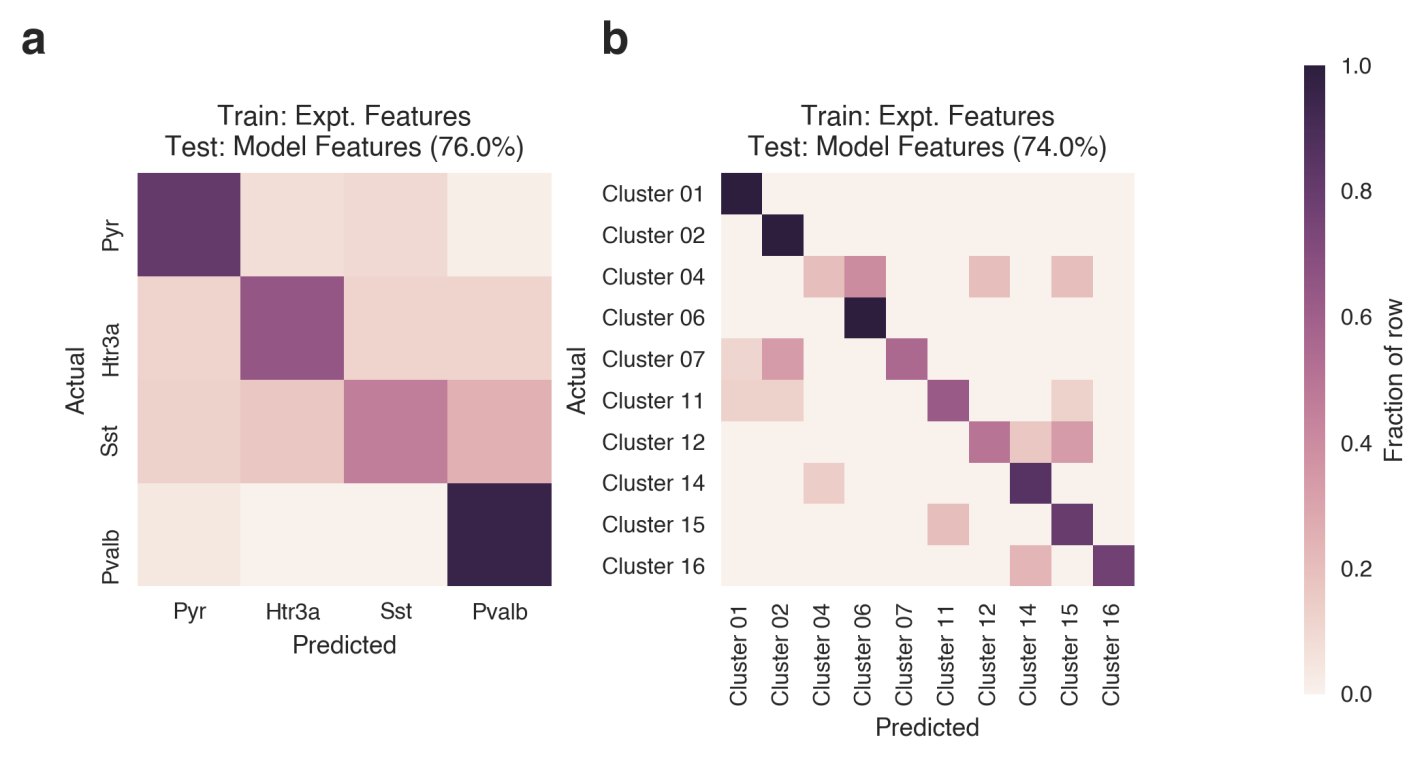


**Supplementary Figure 5:** Prediction of cell classes by training on experimental features and testing on model features. A supervised classifier was trained on twelve features from the experimental data, then tested on the features of the models generated from the same set of cells. Color scale indicates fraction of models in a given row assigned to a given column. (**a**) Prediction of transgenic line-defined cell classes (n=146 cells, see Supplementary Fig. 3). (**b**) Prediction of cell classes defined by unsupervised clustering (n=89 cells, see Supplementary Fig. 4). Note that accuracy scores are generally higher than those presented in Fig. 7 since the scores of the latter were calculated using subsets of the data via five-fold cross-validation.

**Supplementary Table 1:** Features for optimization and minimum tolerances.

| Feature | Tolerance |
| --- | --- |
| Average firing frequency | 0.5 Hz |
| Latency to first spike | 5 ms |
| Average interspike interval (ISI) | 0.5 ms |
| Duration of first ISI | 1 ms |
| Coefficient of variation of ISIs | 0.1 |
| Adaptation index | 0.001 |
| Action potential peak | 2 mV |
| Fast trough depth | 2 mV |
| Slow trough depth | 2 mV |
| Time of slow trough (as fraction of ISI) | 0.05 |
| Action potential width at half-height | 0.1 ms |
| Resting potential | 2 mV |

##

## References

1. Reuveni, I., Friedman, A., Amitai, Y. & Gutnick, M. Stepwise repolarization from Ca2+ plateaus in neocortical pyramidal cells: Evidence for nonhomogeneous distribution of HVA Ca2+ channels in dendrites. *J. Neurosci.* **13,** 4609–4621 (1993).

2. Avery, R. & Johnston, D. Multiple channel types contribute to the low-voltage-activated calcium current in hippocampal CA3 pyramidal neurons. *J. Neurosci.* **16,** 5567–5582 (1996).

3. Randall, A. & Tsien, R. Contrasting biophysical and pharmacological properties of T-type and R-type calcium channels. *Neuropharmacology* **36,** 879–893 (1997).

4. Destexhe, A., Mainen, Z. & Sejnowski, T. Synthesis of models for excitable membranes, synaptic transmission and neuromodulation using a common kinetic formalism. *J. Comput. Neurosci.* **1,** 195–230 (1994).

5. Kole, M., Hallermann, S. & Stuart, G. Single Ih channels in pyramidal neuron dendrites: Properties, distribution, and impact on action potential output. *J. Neurosci.* **26,** 1677–1687 (2006).

6. Adams, P., Brown, D. & Constanti, A. M-currents and other potassium currents in bullfrog sympathetic neurones. *J. Physiol. (Lond.)* **330,** 537–572 (1982).

7. Vervaeke, K., Hu, H., Graham, L. & Storm, J. Contrasting effects of the persistent Na+ current on neuronal excitability and spike timing. *Neuron* **49,** 257–270 (2006).

8. Korngreen, A. & Sakmann, B. Voltage-gated K+ channels in layer 5 neocortical pyramidal neurones from young rats: Subtypes and gradients. *J. Physiol. (Lond.)* **525 Pt 3,** 621–639 (2000).

9. Foust, A., Yu, Y., Popovic, M., Zecevic, D. & McCormick, D. Somatic membrane potential and Kv1 channels control spike repolarization in cortical axon collaterals and presynaptic boutons. *J. Neurosci.* **31,** 15490–15498 (2011).

10. Keren, N., Peled, N. & Korngreen, A. Constraining compartmental models using multiple voltage recordings and genetic algorithms. *J. Neurophysiol.* **94,** 3730–3742 (2005).

11. Liu, P. & Bean, B. Kv2 channel regulation of action potential repolarization and firing patterns in superior cervical ganglion neurons and hippocampal CA1 pyramidal neurons. *J. Neurosci.* **34,** 4991–5002 (2014).

12. Hay, E., Hill, S., Schürmann, F., Markram, H. & Segev, I. Models of neocortical layer 5b pyramidal cells capturing a wide range of dendritic and perisomatic active properties. *PLoS Comput. Biol.* **7,** e1002107 (2011).

13. Köhler, M. *et al.* Small-conductance, calcium-activated potassium channels from mammalian brain. *Science* **273,** 1709–1714 (1996).

14. Magistretti, J. & Alonso, A. Biophysical properties and slow voltage-dependent inactivation of a sustained sodium current in entorhinal cortex layer-II principal neurons: A whole-cell and single-channel study. *J. Gen. Physiol.* **114,** 491–509 (1999).

15. Colbert, C. & Pan, E. Ion channel properties underlying axonal action potential initiation in pyramidal neurons. *Nat. Neurosci.* **5,** 533–538 (2002).

16. Carter, B., Giessel, A., Sabatini, B. & Bean, B. Transient sodium current at subthreshold voltages: Activation by EPSP waveforms. *Neuron* **75,** 1081–1093 (2012).

17. Johnston, D. & Wu, S. *Foundations of cellular neurophysiology*. (MIT Press, 1995).

18. Teeter, C. *et al.* Generalized leaky integrate-and-fire models classify multiple neuron types. *Nat. Commun.* doi: 10.1038/s41467-017-02717-4 (2018).
